# Neonatal Mortality and Prevalence of Practices for Newborn Care in a Squatter Settlement of Karachi, Pakistan: A Cross-Sectional Study

**DOI:** 10.1371/journal.pone.0013783

**Published:** 2010-11-01

**Authors:** Afsheen Ayaz, Sarah Saleem

**Affiliations:** 1 Department of Microbiology, Aga Khan University Hospital, Karachi, Pakistan; 2 Department of Community Health Sciences, Aga Khan University Hospital, Karachi, Pakistan; Kenya Medical Research Institute, Kenya

## Abstract

**Background:**

During the past two decades there has been a sustained decline in child and infant mortality, however neonatal mortality has remained relatively unchanged. Almost all neonatal deaths (99%) occur in developing countries, where the majority are delivered at homes. Evidence suggests that these deaths could be prevented by simple, inexpensive practices and interventions during the pregnancy, delivery and postnatal period. In Pakistan over the last decade extensive efforts have been made by the international donors and government to implement these practices. However, limited attempts have been made to explore if these efforts have made a difference at the grass root level. This study assessed the burden of neonatal mortality and prevalence of practices for newborn care in a squatter settlement of Karachi, Pakistan.

**Methodology/Principal Findings:**

A community based cross-sectional study was performed. A pre-tested structured questionnaire was administered to 565 women who had recently delivered. Information was collected on neonatal morbidity, mortality and practices of women regarding care during pregnancy, child birth and for newborn, till 28th day of birth. Although 70% of women mentioned receiving antenatal care by a skilled provider, only 54.5% had four or more visits. Tetanus toxoid was received by 79% of women while only 56% delivered at a health care facility by a skilled attendant. Newborn care practices like bathing the baby immediately after birth (56%), giving pre-lacteals (79.5%), late initiation of breast feeding (80.3%), application of substances on umbilical cord (58%) and body massage (89%) were common. Most neonates (81.1%) received BCG injection and polio drops after birth. Neonatal mortality rate was 27/1000 live births with the majority of deaths occurring during the first three days of life.

**Conclusion:**

Even after years of efforts by government and nongovernmental sector to reduce newborn morbidity and mortality, inadequate antenatal care, home deliveries and unhealthy newborn care practices are highly prevalent. This leads us to important questions of why practices and behaviors have not changed. Who is responsible and what strategies are needed to bring this change?

## Introduction

Globally there has been a considerable decline in under-five and infant mortality in the past two decades. However neonatal mortality remains relatively unchanged especially in developing countries.[1], [2] Worldwide four million infants die in the first 28 days of life each year: the neonatal period. Three quarters of these neonatal deaths occur in the first week of life, and more than one quarter occur in the first 24 hours after birth.[1], [3], [4] The estimated 3.2 million still births per year globally are not part of this mortality.[5] Almost all of neonatal mortality is observed in developing countries and mostly in babies who are born at homes.[1], [6] The highest neonatal mortality rates are seen in sub Saharan Africa. In Asia the average rates are lower but this region accounts for over 60% of the estimated global total, mainly because of the large population and high fertility rate.[2], [7], [8] Pakistan ranks third, among the ten high burden countries and accounts for 7% of global neonatal deaths.[9]

The major causes of neonatal mortality globally are severe infections (36%, including sepsis/pneumonia {26%}, tetanus {7%}, diarrhea {3%}), preterm birth (28%) and asphyxia (23%).[3] Evidence suggests that a significant proportion of neonatal morbidity and mortality in developing countries could be prevented through inexpensive, simple practices and interventions during pregnancy, delivery and postnatal period. These include tetanus toxoid immunization to mothers, proper nutrition including iron, folate and iodine supplements, clean and skilled care at delivery, newborn resuscitation, prevention of hypothermia, early and exclusive breast feeding, clean umbilical cord care and management of pneumonia and sepsis. [10]–[14] However these interventions cannot produce optimal results unless there is synchronization between the services provided and the reciprocal change in practices and behaviors amongst the recipients. The technical competency for provision of services and bringing behavioral change cannot be over emphasized.

Over the years numerous initiates have been launched globally by international organizations with focus on developing countries to implement these cost effective interventions. In Pakistan the Department of International Development UK (DFID), United States Agency for International Development (USAID), Canadian International Development Agency (CIDA) and Japan International Cooperation Agency (JICA) provided financial support to public and NGO sectors to improve the health of mothers and children in a number of projects expanding over a period of five years or more at a time. The National Programme for Family Planning and Primary Health Care was launched in 1994. This programme aimed to increase the utilization of effective preventive and curative, maternal and child health services at the community level through a network of lady health workers (LHWs). The program covers approximately 55% of Pakistan's population.[15] Saving Newborn Lives (SNL) was initiated by Save the Children USA in June 2000 in 12 countries. In Pakistan it assisted the Ministry of Health in training over 3000 health care providers and 4000 LHWs in maternal and newborn care to improve basic services. Also a behavior change communication strategy considerably helped in reducing neonatal tetanus. Moreover, an SNL assisted intervention trial to test the effectiveness of a newborn care package within the existing system was conducted, which included two main providers, LHWs and traditional birth attendants (TBAs). This trial showed reduction in neonatal mortality by 28% in the study area.[16], [17] Another important project “Pakistan Initiative for Mothers and Newborns” (PAIMAN) was launched in 2004 in 10 predominantly rural districts of Pakistan with the assistance of USAID. The objective of this project too was improved service provision through using clean delivery kits, identification of danger signs and making timely referrals for home deliveries. This was supplemented by increasing awareness and promoting positive maternal and neonatal health behaviors among communities by using mass media and home visits by trained LHWs and community health workers (CHWs).[18], [19] The Expanded program on Immunization was incepted in 1984 and provides coverage against vaccine-preventable diseases to children less than one year of age and tetanus toxoid to pregnant women. The National Nutrition Program and The Women Health Project are some other examples of efforts made by the government of Pakistan.

Data from Pakistan suggests that under-five mortality rates have declined from 117 to 94 per 1000 live births where as the infant mortality rates have declined from 91 to 78 per 1000 live births during 1990–1991 to 2006–2007. However, the neonatal mortality rates have remained almost static from 56 to 54 per 1000 live births.[20]

With this background of extensive efforts made over the years we were interested in assessing the neonatal mortality and practices of women in relation to care during pregnancy, delivery and for newborn in a squatter settlement of Karachi, Pakistan. The purpose was to generate evidence and recommendations to guide policies and interventions to induce behavior change for saving newborn lives.

## Methods

### Ethics statement

Ethical approval of the study was sought from The Aga Khan University Ethical Review Committee. Written informed consent was obtained from every participant.

### Study Design and Population

A community based cross-sectional study was conducted from August to October 2006 in Azam Basti, one of the largest squatter settlements of Karachi. Approximately 40% of the population of Karachi lives in squatter settlements. These settlements are characterized by poverty, lack of education and poor sanitary conditions. The Department of Community Health Sciences of Aga Khan University (CHS/AKU) had operational Primary Health Care programs (PHC) in six of the squatter settlements of Karachi for 12 years (1984–1996). Azam Basti was one of these field sites. This site was purposely selected to facilitate the interview because the residents were familiar with the staff from CHS/AKU. Azam Basti is a low to middle income community and has a population of nearly 50,000. Majority of the residents are Muslims and the area consists mainly of immigrants from upcountry i.e. Punjab and Khyber Pakhtoonkhwah, formerly known as NWFP. A large government run tertiary care hospital is located close to this area and there is also a mid-level secondary care private hospital situated within the community. Still approximately 40% of deliveries are conducted in homes, mostly by TBAs.

Women who gave birth to a live, singleton baby and were in between the 28 to 33 days post delivery period were interviewed. More than one strategy was used to locate the eligible women. A house to house survey was conducted to identify women who had a live birth in 33 days prior to the survey. During survey if women who were pregnant were identified, they were also informed about the study and their consent to participate was obtained. These houses were revisited at 28 to 33 day after the expected date of delivery. Houses which had an infant younger than 28 days were also revisited when the babies reached the age between 28 to 33 days for the interview. Secondly TBAs with good reputation in the community were approached to identify women whom they have delivered during the past 33 days or women who were registered with them for delivery. TBAs were requested to explore the intentions of women to participate in the study. Those women who showed interest were approached by the research team and were enrolled in the study after gaining their written informed consent. Eligible study subjects were interviewed till the required sample size was achieved. A pretested structured questionnaire was used to glean information on neonatal morbidity, mortality and practices of women regarding care during pregnancy, delivery and for newborn till 28th day of birth.

### Variables and Statistical Analysis

#### Care during pregnancy

Antenatal care during pregnancy was dichotomized into “appropriate care” that is women having ≥4 visits from a skilled provider (doctor) as recommended by WHO[21] and “inappropriate care” where women had <4 visits from a skilled provider or had antenatal care from unskilled providers such as TBAs, LHWs or relatives. Information was also gathered on tetanus toxoid vaccination, oral supplementation (iron and folic acid) and consultation for any illness during pregnancy was also determined.

#### Care during delivery

Care during delivery was assessed by the place where the delivery was conducted and the person conducting delivery. Seeking consultation for any complication during delivery was also determined.

#### Newborn care

Information was gathered on the time of the first bath given to baby, type and the time of first feed, cord care, skin application and immunization of the newborn.

Demographic information and information on pregnancy history was also sought. Statistical Package for Social Sciences (SPSS) version 16 was used for data analysis. Descriptive statistics were computed according to the type of the variable. The mean (± standard deviation) was computed for continuous variables while categorical variables were assessed by computing frequencies (%).

## Results

We completed interviews of 565 women aged 18–46 years. The mean age of the women was 27.2 (±4.5) years. Majority (76.5%) were between 21–30 years of age. Most of the women belonged to five major ethnic groups, i.e. Punjabi (38%), Urdu speaking (23.2%), Sindhi (17%), Hindko (7.3%), and Pukhtun (6.7%). Approximately 69% of women received some form of formal education, 36.5% had 1–5 years of schooling, 30.3% had 6–10 years of schooling and only 2.5% had above 10 years of schooling. Mean number of live births a woman had was 2.6 (±1.5). About 26.2% of women were primiparous (one live birth), 63.2% were multiparous (2–4 live births) and 10.6% were grand multiparous (5 or more live births). About 58% of the neonates in the study were males. (Table 1).

**Table 1:** Demographic and reproductive characteristics of the study population (n = 565).

Characteristics	n	%
**Women's age in years**		
Mean (±SD)	27.2(±4.5)	
15–20 years	43	7.6
21–25 years	165	29.2
26–30 years	267	47.3
31–35 years	58	10.3
36 & above	32	5.7
**Ethnicity**		
Punjabi	219	38
Urdu	131	23.2
Sindhi	96	17
Hindko	41	7.3
Pukhtun	38	6.7
Others	40	7.1
**Respondent's Education**		
Illiterate	174	30.8
1–5 yrs of schooling	206	36.5
6–10 yrs of schooling	171	30.3
>10 yrs of schooling	14	2.5
**Parity**		
Mean Parity (±SD)	2.6(±1.5)	
Primiaprous	148	26.2
Multiparous	357	63.2
Grand-multiparous	60	10.6
**Sex of the baby**		
Female	239	42.3
Male	326	57.7

### Care during pregnancy

Majority (70%) of women reported receiving antenatal care from a skilled provider (doctor) during pregnancy however only 54.5% had four or more visits i.e. appropriate antenatal care as recommended by WHO.[21] About 79% of women received tetanus toxoid vaccination during pregnancy and of these 88% had two doses of the vaccine. Almost all women (94.3%) reported taking some kind of micronutrient supplementation during pregnancy but they could not relate the type, dose and number of days they had taken it. Of those who reported taking folic acid only 16% had it during the first trimester of pregnancy. (Table 2) About 22% of women reported some kind of illness at any time during pregnancy. Swelling of legs and face was most common (38%), followed by vomiting (22%), vaginal discharge (14.5%), vaginal bleeding (11.3%), backache (10.5), and high blood pressure (8.1%). Almost all of these women (94%) consulted a doctor for their illness. (Table 2).

**Table 2:** Percentage distribution of Antenatal Care practices.

Characteristic	Frequency	Percentage
**Received appropriate antenatal care** *		
Yes	308	54.5
No	257	45.5
**Received Tetanus Toxoid**		
Yes	445	78.8
No	120	21.2
**Supplementation during pregnancy**		
Yes	533	94.3
No	32	5.7
**Folic acid supplementation**		
Yes	406	71.9
No	159	28.1
**Folic acid during first trimester**		
Yes	88	15.6
No	477	84.4
**Illness during pregnancy**		
Yes	125	22.1
No	440	77.9
**Consultation for illness**		
With doctor	117	93.6
Other than doctor	8	6.4

*Appropriate antenatal care: a woman having ≥4 visits from a skilled provider.

### Care during delivery

Fifty six percent of women delivered at a health care facility i.e. hospital or a birthing clinic by a doctor or a nurse. The women who delivered at their homes (n = 248) mostly had TBAs (73%), and LHWs (22%) to assist them. A small number of women (5%) called for a doctor, nurse or a relative to help them deliver at home. Five percent of women delivered babies through caesarian section. (Table 3).

**Table 3:** Percentage distribution of Natal care practices.

Characteristics	Frequency	Percentage
**Place of delivery**		
Hospital	211	37.3
Birthing clinic	106	18.8
Home	248	43.9
**Person conducting delivery**		
Doctor/Nurse	320	56.7
LHW	57	10.1
TBA	183	32.4
Relative	5	0.9
**Type of delivery**		
Vaginal	536	94.9
Caesarian	29	5.1
**Instrument used in home deliveries**		
Clean	132	53.2
Unclean	1	0.4
Don't know	115	46.4
**Newborn properly** * **covered after birth**		
Yes	560	99.1
No	3	0.5
Don't know	2	0.4

*Properly covered: Newborn covered from head to toe when handed over to mother.

Of those who delivered at home, 53% were aware that a clean instrument from a sealed pack was used to cut the cord .One woman reported that an unclean blade was used. Remaining women were not aware of the instrument used to cut the cord and whether it was clean or not. Almost all women mentioned that their baby was properly wrapped before being handed over to them by the birth attendant. (Table 3)

### Newborn care

According to the WHO's recommendation, bathing the newborn should be delayed for at least six hours after birth.[22] In our sample 56% of neonates were given bath within 6 hours of birth. Of these majority (70%) were bathed within an hour after birth. Early bathing was observed in 87% of babies who were delivered in homes as compared to 31.5% who were delivered at a facility. Eleven babies did not receive bath as they were too ill and died later. (Table 4).

**Table 4:** Percentage distribution of postnatal newborn care practices.

Characteristic	Frequency	Percentage
**Time of first bath after birth**		
<6 hrs of birth	316	55.9
≥ 6 hrs of birth	238	42.1
*Too ill to be given bath	11	1.9
**Received pre-lacteals**		
Yes	449	79.5
No	109	19.3
*Too ill to be fed	7	1.2
**Time of first breast feed**		
≤2 hours of birth	95	16.8
>2 hours of birth	450	79.6
Not fed breast milk	20	3.5
**Breast feeding by 28^th^ day**		
Yes	531	94
No	19	3.4
Expired	15	2.7
**Supplementary feeds**		
Yes	85	15
No	465	82.3
Expired	15	2.7
**Received Immunization**		
Yes	458	81.1
No	93	16.5
#Expired	14	2.5
**Application on the cord**		
Yes	326	57.7
No	225	39.8
#Expired	14	2.5
**Neonatal Massage**Yes	504	89.2
No	47	8.3
#Expired	14	2.5

*Most of these babies died within first three days of life.

#These babies were ill at birth and did not receive immunization, application.

on cord or neonatal massage.

Nearly 80% of newborns received some kind of pre-lacteal feed which included traditional substances like honey (66%), ghutti (herbal paste) (11%), and arq (rose water) (2.5%).Among those who delivered at home 93% received pre-lacteals whereas in institutional births about 71% received pre-lacteals. WHO in the 1980's recommended that breast feeding should be started within an hour of child birth. [23], [24] In our study we found that most of the women (80.3%) initiated breast feeding after 2 hours of delivery. Among home deliveries 92% breast fed their babies after 2 hours of birth whereas in institutional deliveries 76% breast fed after 2 hour of birth. Despite giving pre-lacteals and late initiation, breast feeding was universal in neonatal period; nearly 94% of women were breast feeding by 28th day of delivery. Exclusive breast feeding was common in the neonatal period. Only 15% of women reported giving supplementary feeding. (Table 4)

Most of the neonates (81.1%) received BCG injection and polio drops after birth. More babies who were born in hospitals were vaccinated (86%) as compared to those who were born at homes (75%) (Table 4).

Application of indigenously made substances on the umbilical stump and skin of the baby was a common practice. Fifty eight percent of women used some application on the umbilical cord which included ointment (33%), ghee (saturated oil) (27%), coconut oil (19%), mustard oil (9.5%) , some also applied substances like surma (locally made kohl), clove oil, turmeric and talcum powder on umbilical stump. Body massage to newborns was practiced by nearly 89% of women; substances mostly used included mustard oil (73%) and ghee (15%) (Table 4).

### Neonatal morbidity and mortality

Approximately 28% of neonates (156/565) in our sample developed some kind of illness by the 28th day of birth. The illnesses as mentioned by mothers were fever (45.5%), vomiting (27%), difficulty in breathing (15.4%), and reluctance to feed (13.5%). Few (19%) had seizures, lethargy, low temperature, fluid aspiration, jaundice and cyanosis. Of these 82.1% (128/156) became ill during the first week of life and 88.5% of women sought care from a doctor for the neonatal illness. (Table 5)

**Table 5:** Description of neonatal morbidity and mortality.

Characteristics	Frequency	Percentage
**History of illness**		
Yes	156	27.6
No	409	72.4
**Age at illness**		
1–7 days	128	82.1
> 7 days	28	17.9
**Sex of the ill neonates**		
Male	97	62.2
female	59	37.8
**Sought treatment for illness**		
Yes	148	94.8
No	8	5.1
**Provider**		
Doctor	131	88.5
Others	17	11.5
**Outcome of illness**		
Illness resolved	141	90.4
Expired	15	9.6
**Age at death**		
Early neonatal (1–7 days)	13	86.7
Late neonatal (>7 days)	2	13.3

The neonatal mortality rate in our sample population was 27/1000 live births, where 87% (13/15) babies died within the first three days of life and 67% (10/15) of these deaths were on the first day of birth. Most of the women (11/15) who lost their babies received appropriate antenatal care and delivered at a health care facility. (8 in hospital & 3 in birthing clinic)

## Discussion

Despite numerous efforts made over the past years to improve newborn morbidity and mortality in the country, we found that inadequate antenatal care, home deliveries and unhealthy newborn care practices by women were common in this squatter settlement of Karachi. It seems that the interventions and related health messages implemented over the years have not trickled down to the poor population of urban slums or probably they were not effective in bringing about behavioral change and awareness amongst the communities.

Antenatal care four times during pregnancy from a skilled provider is recommended by WHO since 1994 [21] and is shown to be associated with improved maternal and neonatal mortality [25], [26] Women in our study sample did seek antenatal care from a skilled provider however they did not do it for the required frequency. Also a substantial number of women preferred to deliver at home. Theses lacunae in practices are detrimental to the health of newborns and needs further research to determine, why such decisions are made at the household level. Also we need to change the approach adopted by current programs, which were not sufficient to bring change. The poor performance could be related to either poor service provision or underutilization of services by the users or both.

It was encouraging to observe that 79% of women received tetanus toxoid immunization, and of these 88% had received two doses of the vaccine, which is above the national figure. Taking supplementation during pregnancy was reported by almost every woman; however they were not aware of the type and also could not relate dose and number of days they have taken it. Among those who reported taking folic acid, less than a quarter took in the first trimester, the time where it has the most beneficial effect on the growing fetus.[27], [28] Among the women who delivered at homes, many were not even aware of the instrument being used to cut the cord, leave alone its safety. It is alarming to note this inconsistency in practices and behavior of women. We believe that this is due to health programs being vertical in nature, resources not being shared by various ministries and departments and multiple levels of quality of monitoring and implementation of programs.

Bathing the baby immediately after birth was more common in home deliveries (87%) compared to facility based deliveries (31.5%).In South Asia the baby is considered to be unclean (napak) after birth, this belief promotes immediate bathing of the baby, which in turn makes the newborn vulnerable to hypothermia.[29] One encouraging finding was that 94% of women were exclusively breast feeding their babies by day 28th of delivery. On the other hand, giving prelacteals and delayed breast feeding was also common, both in home and facility based deliveries. WHO coined the idea of baby friendly hospitals in order to support early initiation of breast feeding and to discourage bottle feeding in 1992.[30]Government of Pakistan along with WHO claimed many hospitals as “baby friendly hospitals”, but somehow this good initiative has lost its vigor. Application of substances on the umbilical cord and massage to the newborn was also commonly practiced like in other parts of South Asia. [31], [32] Application on the umbilical cord poses a significant risk of neonatal tetanus. [33] Also not all oils used for neonatal massage are beneficial.[34], [35] Bringing about a change in such harmful practices requires an understanding of the deep rooted concepts of physical and spiritual benefits gained by such practices. An understanding of such phenomena and related interventions to bring behavior change in communities have shown reduction in maternal and newborn morbidity and mortality in various regions of developing world.[11], [13], [14], [36], [37] From Pakistan limited information is available on efforts made to bring change in practices[17], [38] however these were implemented on small scales and there is no evidence that strategies have been scaled up to larger populations. However the scaling up of such interventions remains a big challenge for countries with limited resources as they are costly, labor intensive and time consuming. Having said this, we believe that change in practices and a behavior is the need of the day for better health and with whatever scarce resources available; we need to rethink our priorities and formulate strategies accordingly.

Quality of care at the level of hospitals and birthing clinics is another important factor for the survival of neonates. An alarming paradox for neonatal mortality observed in our study was that most of the women whose neonates died received appropriate antenatal care and majority of them delivered in a health care facility, but the health care system they sought possibly failed to respond to their needs. Some of the evidence from Pakistan also suggests a failure of Pakistani health facilities to offer quality essential and comprehensive obstetric and newborn care.[39] The public and private sector needs vigilant monitoring and ongoing research to improve the quality of health service provision.

Some methodological constraints need to be considered when interpreting the results. This study is based on reported newborn care practices and not on actual observation and hence is subject to recall and response bias. The generalizibility of the study is limited as we have sampled subjects from one urban squatter settlement of Karachi. However the urban slums are not very different from others and our results reasonably represent the situation of the urban squatter settlements of Karachi. Due to the sampling strategy adopted we might have missed newborns that died, resulting in underestimation of the neonatal mortality rate.

Pakistan needs more scientific evidence on practices of both health service providers and users to identify what works and what does not in the local context. It is equally important to monitor change in practices and behaviors to guide interventions. Strategies to scale up such interventions should also be tested and implemented in a phased approach in partnership with government and donor agencies.

### Conclusion

Even after years of efforts from the government and nongovernmental sectors to reduce newborn morbidity and mortality, inadequate antenatal care, home deliveries and unhealthy newborn care practices are highly prevalent. This leads us to important questions of why practices and behaviors have not changed at the grass root level. Who is responsible and what strategies are needed to bring this change?
